# Prognostic significance of CXCR4 expression in acute myeloid leukemia

**DOI:** 10.1002/cam4.2535

**Published:** 2019-09-13

**Authors:** Wen Du, Cong Lu, Xinyun Zhu, Dong Hu, Xiangjun Chen, Juan Li, Wei Liu, Jiang Zhu, Yanli He, Junxia Yao

**Affiliations:** ^1^ Center for Stem Cell Research and Application Union Hospital Tongji Medical College Huazhong University of Science and Technology Wuhan China; ^2^ Neonatal Screening Center The Third Affiliated Hospital of Zhengzhou University Zhengzhou China

**Keywords:** AML, clinical characteristics, CXCR4, FLT3‐ITD mutant

## Abstract

**Background:**

CXCR4 chemokine receptors play an important role in leukemia proliferation, extramedullary migration, infiltration, adhesion, and resistance to chemotherapy drugs.

**Methods:**

The CXCR4 expression by flow cytometry in 122 acute myeloid leukemia (AML) patients between 2010 and 2014 was analyzed.

**Results:**

The expression of CXCR4 in AML‐M4/M5 was found to be significantly higher than that of other subtypes according to both FAB subtype and WHO classification. The FLT3‐ITD mutant was significantly higher in high CXCR4 expression group (*P* = .0086). Our data also showed that CXCR4 expression was correlated with CD64 expression. Low CXCR4 expression on AML cells was associated with better prognosis, and the median overall survival (OS) for low CXCR4 expression patients was 318 days, compared with 206 days for patients with high CXCR4 expression (*P* = .045). Multivariate analysis revealed that CXCR4 expression, age, and extramedullary infiltration were independent prognostic factors.

**Conclusions:**

Our study demonstrated that CXCR4 expression in AML was an independent prognostic predictor for disease survival that could be rapidly and easily determined by flow cytometry at disease presentation.

## Graphical abstract

We found that the expression of CXCR4 in AML‐M4/M5 subtype was significantly higher than that of other subtypes. We found low CXCR4 expression on AML cells correlated with a better prognosis, resulting in a longer overall survival. We conclude that CXCR4 expression in AML is an independent prognostic predictor for disease survival that can rapidly and easily be determined at disease presentation.

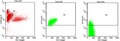

## 1 INTRODUCTION

CXCR4, CXC chemokine receptor 4 (CD184), belonging to specific G protein‐coupled receptors, is the specific receptor of the chemokine matrix cell derivative‐1 (SDF‐1, CXCL12), and has a high affinity with the ligand.^1^ It is expressed in most tissues and organs in the body and participates in various physiological mechanisms. CXCR4 is expressed not only on the surface of hematopoietic stem cells, but also expressed on leukemic blasts and leukemia cell lines. It plays an important role in leukemia proliferation, extramedullary migration, infiltration, adhesion, and resistance to chemotherapy drugs.^2^, ^3^, ^4^

Acute myeloid leukemia (AML) is a malignant proliferative disease of myeloid hematopoietic stem cells and primitive cells with high heterogeneity.^5^, ^6^, ^7^, ^8^ The development and prognosis of AML are correlated with the factors such as the number of leukocytes in peripheral blood, cytogenetic abnormality, lactic dehydrogenase (LDH) level, body state, and others.^3^ Now, there have been increasing studies showing that the high expression of CXCR4 is a poor prognostic factor in some solid tumors and leukemia.^3^, ^9^, ^10^

*FLT3* is a cytokine receptor that is expressed on the leukemic blasts in acute leukemia. AML with a *FLT3* internal tandem duplication (*FLT3‐ITD*) mutation has a generally poor prognosis.^11^, ^12^, ^13^, ^14^, ^15^ Some research investigated the correlation of CXCR4 and its ligand's expression with the clinical outcome in patients with AML.^16^, ^17^ Rombouts EJ et al have reported that CXCR4 expression levels were elevated in AML patients with *FLT3‐ITD* mutations,^18^ but Mannelli F's study pointed out that *FLT3‐ITD* mutations had no effect on CXCR4 expression level.^19^ Given the controversy study results, we studied CXCR4 expression in AML and its relationship with clinical characteristics, molecular biology, therapeutic reactivity, and prognosis. In addition, we investigated the relationship between *FLT3* gene and CXCR4 tested in the risk stratification model of AML.

## 2 PATIENTS, MATERIALS, AND METHODS

### 2.1 Patients

From June 2010 to December 2014, 122 patients with AML were enrolled in this study. Bone marrow samples of patients with AML were obtained after informed consent at the time of diagnosis. The diagnosis was based on MIC (Morphologic, Cytochemical, and Immunophenotype) criteria. Patients' characteristics were displayed in Table 1. Induction therapy plan was chosen based on patient's age. For patients younger than 60 years, DA (daunorubicin and cytarabine）regimen, HA (Homoharringtonine and cytarabine, three sharp cedar ester alkali and cytarabine) regimen, or MEA (mitoxantrone, etoposide, and cytarabine) were suggested and taken. Patients older than 60 years old were suggested and treated with CAG (accra toxin, cytarabine, and G‐CSF) regimen. Medium dose Cytarabine was mainly used in the consolidation cycle. This study was approved by the Research Ethics Committee of Union Hospital, Tongji medical College, Huazhong Science & Technology University.

**Table 1:** Basic clinical characteristics of all enrolled patients (n = 122)

Basic clinical characteristics	Value
Age median (range)	43(3‐76) years
(≥60Y, <60Y)	(16/106)
Gender (males/females, n)	72/ 50
Hemoglobin, median ( range)	72 (54‐129) (g/L)
Leukocyte count, median ( range)	29.64(0.97‐384.5) (×10^9^/L)
Platelet count, median ( range)	38(7‐162) (×10^9^/L)
Blasts (%), median ( range)	71% (20%‐98%)
FAB classification, n	
M0/M1	13
M2	58
M3	14
M4/M5	34
M6	1
Unclassified	2
WHO classification, n	
AML with recurrent genetic abnormalities	
t(8:21)/*ETO*	5
inv(16)/t(16;16)/*CBFB‐MYH11*	6
t(15;17)/*PML‐RARα*	7
11q23/*MLL*	1
t(6;9)/*DEK*	1
*NPM1*	6
*CEBPA*	6
AML with myelodysplasia‐related changes (MRC)	13
AML‐NOS, n	
M0/M1	3
M2	11
M4/M5	9
Unclassified	54
*FLT3* status, n	
Mutated	40
Wild type	65
NA	17

### 2.2 Flow cytometry

Routing standard immunophenotyping was performed for each BM sample, including the expression of CD14, CD64, CD117, and CD34.

EDTA‐anticoagulated fresh bone marrow aspirates from 122 patients were analyzed. Surface and intracellular antigen detection was performed on fresh bone marrow samples within 2 hours by multicolor flow cytometry. Blast cells were gated according to their CD45/SSC properties.

For surface antigen staining, 1 × 10^6^ cells were incubated for 30 minutes with 10 μL of appropriately diluted monoclonal antibody conjugates. APC‐conjugated antibodies were used to detect CXCR4, in combination with CD45‐PerCP. After removing the red blood cells by lysis and washing in phosphate‐buffered saline (PBS), concentrating, and removing supernatant, the cell suspension was treated with 100‐μL PBS for 15 minutes and processed to flow cytometry analysis. Control samples were incubated with isotype control antibodies. Data were acquired using a FACS Calibur flow cytometry (Becton Dickinson). FSC/SSC combined with CD45/SSC 2‐d scatter plot was gated to delineate the abnormal cell group. Data were analyzed by FCS Express V3 software. Surface antigen expression was assessed as percentage of positive cells.

### 2.3 Detection of *FLT3*, *NPM1*, and *CEBPA* mutations

*FLT3* gene and *NPM1, CEBPA* gene mutations were all amplified by reverse transcriptase polymerase chain reaction (RT‐PCR). The products were screened by agarose gel electrophoresis. For the screening of *FLT3* mutations, we amplified genomic DNA corresponding to exons 14 and 15. Primer sequence and fragment size of *FLT3* were: Upstream sequence (14 exon region) 5ʹ‐GCAATTTAGGTATGAAA GCCAGC‐3ʹ and downstream sequence (15 exon region): 5ʹ‐CTTTCAGCATTTTGA CC‐3ʹ.^19^ For the *NPM1* mutations corresponding to exon 12, primers were *NPM1*‐F: 5ʹ‐TTAACTCTCTGGTGGTAGAATGAA‐3ʹ and *NPM1*‐R: 5ʹ‐CAAGAC TATTTGCCATTCCTAAC‐3ʹ.^20^ As for the *CEBPA* mutations, the 3ʹ coding region of the *CEBPA* gene was amplified using forward primer 3 and reverse primer 8. The N‐terminus was amplified by forward primer 1 and reverse primer 5 as previously described.^21^ Procedure was described as extracting RNA, RNA reverse transcription, PCR reaction system, and agarose gel identification. PCR products were resolved on 3% agarose gel. The gel was removed from the electrophoresis tank and put into Bio‐Rad ChemiDoc XRS + gel imaging system (Bio‐Rad, Hercules, CA) for observation and analysis.

### 2.4 Karyotype analysis

Conventional cytogenetic analysis was performed on bone marrow cell by G‐banding pattern. The chromosomal aberrations were described according to the International System for Cytogenetic Nomenclature (ISCN) 2009.^22^

### 2.5 Statistical analysis

SPSS18.0 software (SPSS, Chicago, IL) was used for the statistical analysis. Associations between clinical factors in group comparisons were performed by the Mann‐Whitney nonparametric U test and Kruskal‐Wallis test or Fisher exact tests. The correlation of CXCR4 with other antigens was analyzed by simple linear regression analysis. For the survival analysis, the Kaplan‐Meier survival curves and the log rank test were used. Complete remission (CR) was defined as <5% blasts in bone marrow aspirates, peripheral blood lacking leukemia blasts and restoration of peripheral blood counts. Relapse‐free survival (RFS) was measured from CR date to relapse or last follow‐up. The overall survival (OS) was calculated from the diagnosis to the last observation or death. RFS and OS were analyzed by the Kaplan‐Meier method. Univariate and multivariate analyses on categorized data were performed using Cox proportional hazards mode, which were fitted to evaluate the effects of patient characteristics on RFS and OS. In all evaluations, *P* value below .05 was considered significant.

## 3 RESULTS

### 3.1 Patient characteristics

From February 2010 to December 2014, 122 adult AML patients in Union Hospital were studied. Their basic characteristics are shown in Table 1.

### 3.2 Expression of CXCR4 by flow cytometry

**Figure 1:**
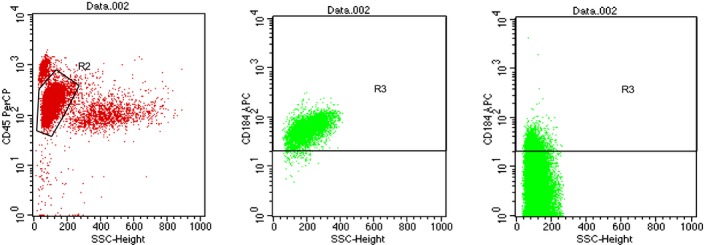
Representative examples of the flow cytometric analysis of CXCR4 expression on AML blast

**Figure 2:**
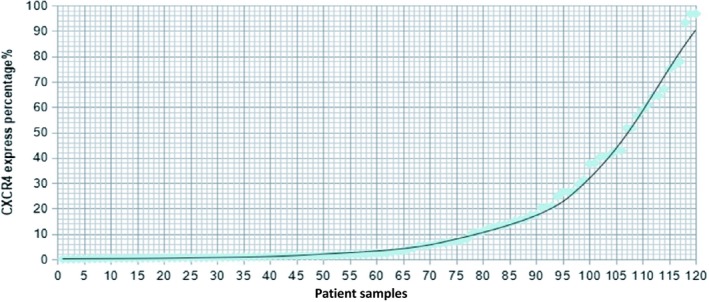
The distribution of CXCR4 expression of the enrolled 122 patient samples

We examined CXCR4 surface expression in AML blasts of the 122 samples. Representative samples of high or low CXCR4 expression by AML cells are displayed in Figure 1. Using flow cytometry, we observed a continuous and heterogeneous in the levels of CXCR4 expressed by AML blasts. The percentage of CXCR4 expression was ranging from 0.03% to 96.75% with a median value of 2.25% (Figure 2).

**Figure 3:**
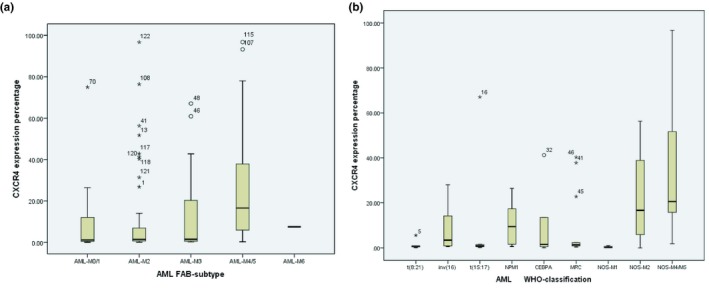
The distribution of CXCR4 expression in AML patients according to (A) FAB‐subtype and (B) WHO classification

By analyzing the CXCR4 expression in each AML subtype, our results showed that the CXCR4 expression in patients with AML M4/M5 subtype was higher than any other subtypes, with significant statistical difference (*P* = .001; Figure 3A). Our data showed that CXCR4 expression was increased significantly in AML‐NOS (M4/M5) subtype (*P* = .026; Figure 3B) in 68 patients who had complete MICM results based on WHO classification.

### 3.3 The association between CXCR4 expression and the clinical characteristics in AML patients

We chose 3.84% as the optimal cut‐point based on the median value and *FLT3‐ITD* mutation status. We displayed all the 122 patients' characteristics for each of the two different CXCR4 expression groups (high or low). We did not find any statistical differences of CXCR4 expression in relation to gender or age. For patients with extramedullary infiltration observed at first visit, we noticed higher extramedullary infiltration rate and failure to CR in patients with higher CXCR4 expression (group A) when compared with patients with low CXCR4 expression (group B), which showed a significant statistical difference (*P* < .05). But there were no significant statistical differences in white blood cell (WBC) count, hemoglobin (Hb), platelet (Plt) count, and blast percentage between the two groups (Table 2).

**Table 2:** Comparison of clinical features between high and low CXCR4 group

Clinical data	Group A （≥3.84%）	Group B (<3.84%)	*P* value
	(n = 33)	(n = 51)	
WBC (1 × 10^9^) (median)	40.03	22.7	.263
Hb (g/L) (median)	81.5	69	.305
Plt (1 × 10^9^) (median)	47	32.5	.400
Blast percentage (%)	71	72	.646
CR, n			
Yes	13	32	.036*
No	20	19	
EI, n			
Yes	8	7	.048*
No	7	23	
*FLT3* status, n			
Wild type	22	39	.0086*
Mutated	24	14	
*NPM1* status, n			
Wild type	16	32	.221
Mutated	4	3	
*CEBPA* status, n			
Wild type	23	36	.842
Mutated	3	4	

* Indicates statistical difference, *P* < .05.

### 3.4 The relationship between CXCR4 expression, *FLT3‐ITD, NPM1, and CEBPA* mutations

In order to be able to evaluate the correlation between CXCR4 expression and *FLT3* mutation, 105 of all the AML samples were analyzed for *ITDs* of the *FLT3* gene. Forty *FLT3* mutations were detected among 105 acute leukemia cases with mutation frequency of 38.10%.

We performed a Fisher exact test and K‐S test to analyze the correlation of the 99 cases who were both detected for *FLT3‐ITDs* mutation and CXCR4 expression. Analysis of flow cytometric data showed that the CXCR4 expression was increased in *FLT3‐ITD* AML (10.48%, 0.55%‐74.98%) when compared with *FLT3/wt* AML (1.86%, 0.03%‐67.05%, *P* = .023). The number in high expression group with *FLT3‐ITD* mutant was distinctly higher than that in low group (*P* = .0086, Fisher exact test) (Table 2). In addition, we also analyzed the relationship between *NPM1* or *CEBPA* mutation status and CXCR4 expression. Focusing on seven *NPM1*‐mutated AML patients and seven *CEBPA*‐mutated AML patients, we compared the CXCR4 expression with the wild‐type group. Data showed that there was no significant difference between these gene mutations and CXCR4 expression which was likely due to small sample size (Tables 2 and 3).

**Table 3:** CXCR4 expression according to *FLT3‐ITD*, *NPM1*, and *CEBPA* mutations

Mutation	CXCR4 expression (median, range)		*P* value
	Mutated	Wild type	
*FLT3‐ITD*	10.48% (0.55%‐74.98%)	1.86% (0.03%‐67.05%)	.023*
*NPM1‐mut*	3.84% (0.60%‐26.41%)	1.33% (0.01%‐67.05%)	.423
*CEBPA‐mut*	3.03% (0.55%‐41.23%)	1.52% (0.03%‐96.75%)	.783

* Indicates statistical difference, *P* < .05.

### 3.5 The relationship between CXCR4 expression and karyotype

Among the 66 patients who had completed chromosomal analysis, there were 34 patients with normal karyotype and 32 patients with abnormal karyotype, respectively. The CXCR4 expression in normal karyotype group was significantly increased (14.98%, 0.26%‐96.67%, *P* < .001) while comparing with the cut‐point value (3.84%). It was hard to look into each abnormal karyotype separately.

### 3.6 CXCR4 expression was correlated with CD64 expression

The relationship between the CXCR4 expression and stem/progenitor cell differentiation antigen CD34 and CD117 was studied. Data showed that CXCR4 expression was in weak correlation (Pearson correlation *r* = .278, *P* = .023) with CD34, but had no correlation with CD117 (*r* = .152, *P* = .067). In the previous analysis, our data showed that CXCR4 expression significantly increased in M4/M5 subtype, so we analyzed the correlation between CXCR4 expression and mononuclear antigen CD14 and CD64 expression. Data showed that CXCR4 and CD64 expressions have certain linear correlation (*R* ^2^ = .818, *P* < .001). Whereas, it was uncorrelated with CD14 expression (*R* ^2^ = .305, *P* = .008).

### 3.7 High CXCR4 expression on AML cells was associated with a decreased overall survival

**Figure 4:**
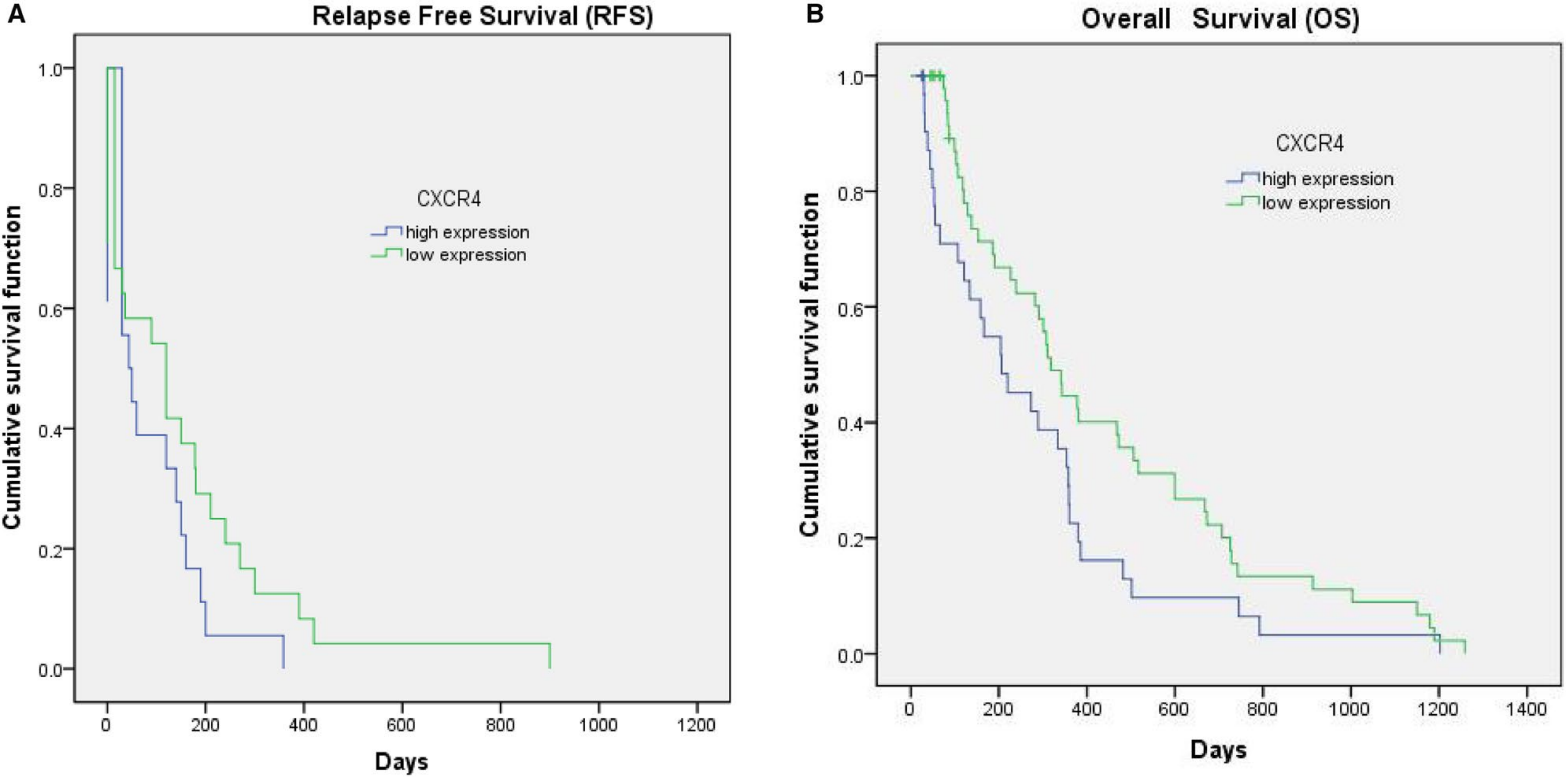
(A) Relapse‐free survival and (B) Overall survival in AML patients according to CXCR4 expression below or above‐equal to the cut‐point

To determine the prognostic impact of CXCR4 expression on AML, we evaluated the OS and RFS of all included prognostic parameters using the Kaplan‐Meier procedure. We conducted a follow‐up of 84 inpatients from February 2014 to February 2018. Among them, eight patients were lost during follow‐up (loss rate was 9.5%). The high level of CXCR4 expression was correlated with a reduced OS (Figure 4). Despite patients in high CXCR4 expression group would be more likely to relapse than those in low expression group, it did not show significant difference in RFS. The significance of Kaplan‐Meier curves of OS on CXCR4 expression and other factors was verified by the log rank test (Table 4). In patient older than 50 years, EI and positive *FLT3‐ITD* mutation also were correlated with a reduced OS.

**Table 4:** Correlation between OS and some clinical‐biological features

Prognostic marker	Significance	*P* value, log rank
Age (Over 50 years or below)	Yes	.006*
WBC (above 20 × 10^9^/L or below)	No	.325
CR (achieving complete remission or failure)	No	.107
EI (Accompanied by extramedullary infiltration or not)	Yes	.025*
*FLT3* (mutant/wild)	Yes	.010*
CXCR4 high/low	Yes	.045*

* Indicates statistical difference, *P* < .05.

Median RFS time in high expression group was 50 days (0‐458 days), meanwhile, median RFS time in low expression group was 168 days (0‐900 days). Data showed that high expression group had a shortened RFS time than low group but there was no statistically significant difference (Log Rank Mantel‐Cox, *P* = .117). For OS time, the median OS time of high group was 206 days (25‐1202 days), which was significantly decreased (Log Rank Mantel‐Cox, *P* = .045) when comparing with the medium OS time in low group ( 318 days ,45‐1259 days).

### 3.8 CXCR4 high expression was an independent risk factor for prognosis in AML patients

To further confirm the observed correlation between disease outcome and CXCR4 expression, we performed a multivariate Cox regression analysis. Gender, age, WBC count, CXCR4 expression, remission after initial chemotherapy (CR), extramedullary infiltration (EI), gene mutation, and cytogenetic abnormalities were included to identify factors affecting the survival of the patients. As a result, the total survival time was not significantly affected by gender, CR, *FLT3*‐mutated, and cytogenetic abnormalities in multivariate Cox regression analysis. Age older than 50 years (*P* = .003, HR = 3.067, 95% confidence interval, 1.372‐6.855), WBC higher than 20 × 10^9^/L (*P* = .016, HR = 2.430, 95% confidence interval, 1.123‐4.619), associated with EI (*P* = .015, HR = 2.575, 95% confidence interval, 0.611‐4.156), and unfavorable gene mutation (*P* = .013, HR = 1.981, 95% confidence interval, 0.843‐4.652) were considered for risk factors that result in reduced OS. It was also showed that CXCR4 high expression was an independent risk factor for total survival time in patients with AML (*P* < .001, HR = 4.422, 95% confidence interval, 1.471‐12.881).

## 4 DISCUSSION

CXCR4 expression was heterogeneous and continuous. We examined for CXCR4 surface expression in AML blasts cells in 122 patient samples, and the expression level was 0.03%‐96.75%, which was consistent with the previous studies. Patients in each AML subtype were divided into high expression and low expression group according to the cut‐point 3.84%. Our study demonstrated that the expression of CXCR4 by flow cytometry in AML‐M4/M5 subtype was significantly higher than that of other subtypes among more than 100 cases. We got the same results in AML according to the WHO classification. In addition, we found that CXCR4 was an independent factor of poor prognosis. In patients with inv (16), the CXCR4 level was significantly lower than patients with AML with monocytic differentiation. Furthermore, we showed that CXCR4 expression was significantly correlated with CD64 expression. We proposed CXCR4 expression detected by flow cytometry could be used as a prognostic marker for AML‐M4/M5. Gao et al^23^ also found that CXCR4 expression in AML‐M4 and M5 subtypes was higher than in AML‐M2 and M3 subtypes through immunohistochemistry, which was consistent with our study.

In previous studies, AML patients with high expression of CXCR4 were reported to have a high frequency of *FLT3* gene mutations of *ITD* type. In vitro experiments showed that overexpression of constitutively activated *FLT3‐ITD* mutants in Ba/F3 cells also activated SDF‐1 signaling.^24^ Therefore, we studied the relationship between CXCR4 expression and *FLT3‐ITD* mutation. The results showed that the level of CXCR4 expression was related to the presence of *FLT3‐ITD* mutations in leukemia blasts. The CXCR4 expression percentage with *FLT3‐ITD* mutant was significantly higher than the group with wild type, consistent with previous research.^18^ This suggests that *FLT3‐ITD* mutation and CXCR4 may have possible interaction, such as *FLT3‐ITD* mutation can activate CXCR4 shift signal, and stromal cells cocultured with *FLT3‐ITD* leukemia cell can weaken *FLT3‐ITD* mutation inhibitors for cell apoptosis. Further research is needed on the relationship between the two signal pathways.

In this study, we investigated the expression of CXCR4 in relation to the clinical outcome of patients with AML.^25^, ^26^ In the AML prognostic group, cytogenetically normal (CN)‐AML was classified as a medium prognostic group, but this type of AML showed heterogeneity in terms of prognosis, and some of them still had poor outcome. In our study, we found that the CXCR4 expression in normal karyotype AML patients was higher than that of the cut‐point value, which may be an explanation for the poor prognosis of patients with normal karyotype. Sergei et al^27^ studied the expression and prognostic significance of CXCR4 in AML patients with normal karyotype and without *FLT3* gene mutation. The results of our study suggest that high CXCR4 expression is still a risk factor for prognosis in normal karyotype AML patients, and its role may be independent.

For the patients with extramedullary infiltration observed at first visit, we noticed higher CXCR4 expression group accompanied with higher extramedullary infiltration rate. We speculated that CXCR4 and its ligands might be important regulatory factors in the mechanism of AML bone marrow infiltration. Voerman et al^17^ confirmed that the migration ability of leukemia cells was positively correlated with the surface expression rate of CXCR4 and the chemotaxis of SDF‐l/CXCR4. SDF‐1 is highly expressed in bone marrow, but also can be produced by other extramedullary tissues such as liver, spleen, and brain, promoting extramedullary migration of leukemia cells. Crazzolara et al^28^ also proved that the increased expression of CXCR4 in leukemia cells indicated the extramedullary organ infiltration of leukemia cells.

In this study, we confirmed that CXCR4 overexpression predicted poor prognosis in AML patients. Patients with lower CXCR4 expression had a significantly longer OS, which was similar to earlier study results.^18^, ^19^, ^29^, ^30^ Ponomaryov et al^31^ observed an increased expression of SDF‐1 in the bone marrow following with DNA‐damaging agents (ionizing radiation, cyclophosphamide, and 5‐fluorouracil [5‐FU]), which resulted in an increase in CXCR4‐dependent homing to the bone marrow and consequently facilitated engraftment of hematopoietic stem cells. In our study, we did not find any correlation between CXCR4 expression and RFS, which may be due to an inexact observation time record of recurrent disease. Moreover, CXCR4 is a prognostic marker that is independent of other classical factors such as age, leukocytosis, *FLT3* mutant, and extramedullary infiltration. However, to assert the prognostic value of CXCR4 as an independent marker, study in larger series of patients should be necessary.

In addition, we found that the patients in CR with higher CXCR4 expression (13/33) decreased significantly compared with the lower group (32/51), and our data showed that high CXCR4 expression might prevent the complete remission of AML patients after primary chemotherapy. We infer that SDF‐1/ CXCR4 axis may help the leukemia cells escape from the attack of the chemotherapy drugs by intrinsic immune regulation, including promoting the proliferation and secretion of cytokines to inhibit cell apoptosis, decreasing the sensitivity to chemotherapy. Dunussi Joannopoulos et al^32^ found that SDF‐1 could regulate immune mechanisms such as tumor growth and immune tolerance in vivo.

In conclusion, CXCR4 is a valuable prognostic marker in AML and it is easy to be measured by flow cytometry. It also can be combined with other antibodies to establish risk‐adapted strategies and could be a potential candidate for targeted therapy. Detecting the expression of CXCR4 may also prompt the presence of extramedullary infiltration. All these findings suggest that CXCR4 could be an important biomarker for the diagnosis and prognosis of AML patients. It would also be especially useful in the risk assessment of AML patient with normal karyotype.

## CONFLICT OF INTEREST

All authors declare that they have no conflict of interests.

## AUTHOR CONTRIBUTIONS

J. Yao and W. Du conceived and coordinated the project. X. Zhu and D. Hu collected the tissue samples. X. Zhu, W. Du, W. Liu, and J. Li performed the flow cytometry analysis. D. Hu, J. Zhu, and C. Lu performed the gene detection. X. Chen and Y. He made and provided the chromosomal analysis. C. Lu and D. Hu provided data acquisition clinical materials and follow‐up data. C. Lu performed the data analysis. C. Lu and J. Yao wrote the manuscript with inputs from all co‐authors. J. Yao supervised the project.

## ETHICAL APPROVAL

All procedures performed in studies involving human participants were in accordance with the ethical standards of the institutional and/or national research committee and with the 1964 Helsinki declaration and its later amendments or comparable ethical standards.

## INFORMED CONSENT

Informed consent was obtained from all individual participants included in the study.

## Data Availability

I confirm that my article contains a Data Availability Statement even if no data are available (list of sample statements) unless my article type does not require one. I confirm that I have included a citation for available data in my references section, unless my article type is exempt.
